# Supplementary material for: THC and CBD Fingerprinting of an Elite Cannabis Collection from Iran: Quantifying Diversity to Underpin Future Cannabis Breeding
Source: Plants (Basel). 2022 Jan 4;11(1):129. doi: 10.3390/plants11010129 (PMC8747537; doi:10.3390/plants11010129)
Supplement: Supplementary file 1 [file plants-11-00129-s001.zip › plants-1467212-supplementary.pdf]

Supplementary Information

THC and CBD fingerprinting of an elite cannabis collection from Iran: quantifying diversity to underpin future cannabis breeding

Mahboubeh Mostafaei Dehnavi, Ali Ebadi, Afshin Peirovi, Gail Taylor, and Seyed Alireza Salami

Supplementary Tables

Table S1. The information of 161 cannabis genotypes analyzed and particular climatic and geographical conditions.

Province	region	Population ID	Individual number	Elevation (m)	Longitude (E)	Latitude (N)	Wind Speed	Avg. annual temp. (C)	Avg. annual min temp. (C)	Avg. annual max temp. (C)	Avg. annual relative humidity	Avg. annual minimum relative humidity	Avg. annual maximum relative humidity	Sunshine duration	Dew Point	Avg. annual precipitation (mm)
Zanjan	Abhar	Abh-01	9	1543	49°03'	36°28'	2.80	12.39	5.98	18.85	51.85	33.32	71.95	8.01	1.003	301.31
Arak	Arak	Ark-01	9	1722	49°42'	34°04'	1.61	14.04	7.27	20.99	45.49	29.49	63.20	8.12	-0.22	297.75
Ardabil	Ardabil	Ard-01	6	1339	48°29'	38°24'	3.81	9.12	3.23	15.68	73.16	52.68	90.51	6.95	3.78	273.67
Kerman	Bam	Bam-01	10	1068	58°14'	29°09'	3.07	23.74	17.98	29.89	25.26	15.56	36.79	9.17	0.29	54.05
Kordistan	Baneh	Ban-01	10	1503	45°53'	35°59'	3.97	14.26	8.81	18.94	44.71	32.44	59.05	7.94	-0.16	660.88
South Khorasan	Boshrouyeh	Bsh-01	10	881	57°43'	34°03'	1.44	21.07	11.53	27.16	34.27	23.30	50.65	8.48	1.81	79.98
Khuzestan	Dezful 1	Dez-01	9	144	48°42'	32°38'	1.35	24.56	16.88	32.54	48.43	28.32	67.81	7.61	10.36	389.40
Khuzestan	Dezful 2	Dez-02	8	144	48°42'	32°38'	1.35	24.56	16.88	32.54	48.43	28.32	67.81	7.61	10.36	389.40
Kerman	Kerman	Krmn-01	7	1761	56°58'	30°15'	2.84	17.01	7.56	25.36	31.68	17.22	50.05	9.17	-2.95	123.43
Kermanshah	Kermanshah	Krsh-01	7	1389	47°03′	34°19′	2.48	15.51	6.99	23.60	43.19	24.93	63.06	7.98	-0.27	402.63
Hamadan	Nahavand	Nhv-01	8	1666	48°25′	34°15′	2.25	14.72	6.56	21.28	45.36	29.58	64.64	8.80	0.85	385.29
West Azerbaijan	Naqadeh	Naq-01	7	1324	45°22'	36°57'	2.68	14.08	5.90	19.33	55.07	40.87	74.16	8.05	3.98	323.95
West Azerbaijan	Piranshahr	Pir-01	7	1572	45°08'	36°42'	2.24	13.28	6.99	18.38	52.18	36.77	70.55	7.88	1.91	640.61
Qazvin	Qazvin	Qzv-01	10	1315	49°86'	36°47'	1.57	13.91	7.19	21.62	52.41	30.56	74.66	8.19	2.46	311.85
Khuzestan	Ramhormoz	Rmhz-01	6	179	49°59'	31°27'	1.99	27.67	19.96	33.20	35.93	24.58	50.90	8.56	7.68	280.49
Hamadan	Samen	Sam-01	6	1858	48°70'	34°20'	2.93	13.53	6.22	20.61	44.18	27.14	63.21	8.48	-0.86	324.79
Kurdistan	Sanandaj	San-01	9	1464	46°99'	35°31'	1.99	14.00	6.12	22.25	47.40	25.83	70.73	8.22	0.04	375.10
Kurdistan	Saqez	Sqz-01	7	1480	46°26'	36°24'	2.26	11.20	2.77	18.94	53.74	34.87	73.73	8.17	0.09	439.37
Kerman	Sirjan	Sir-01	6	1754	55°68'	29°43'	2.58	17.81	9.47	25.36	34.56	20.07	52.13	9.37	-0.31	138.52
Sistan and Baluchestan	Zahedan	Zah-01	10	1352	60°86'	29°49'	3.21	19.30	10.89	27.11	28.88	16.00	28.9	9.26	-2.29	73.58

Supplementary Figures

(a)

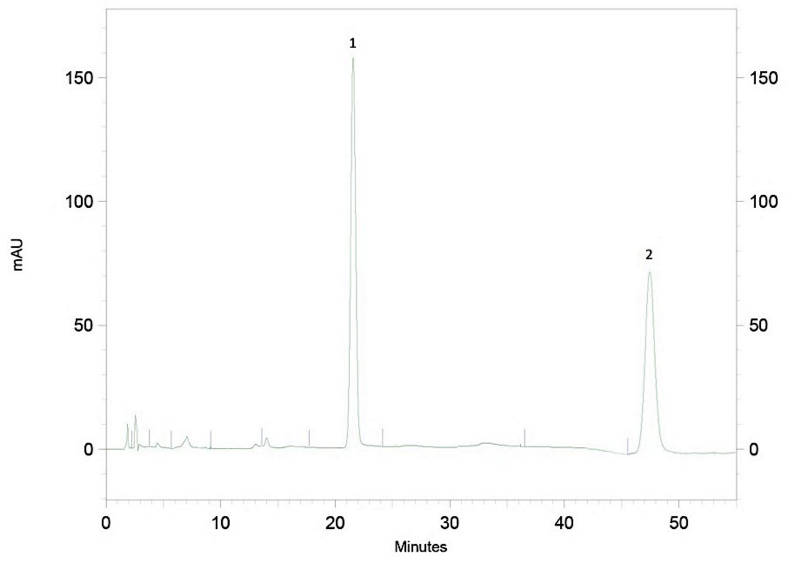

(b)

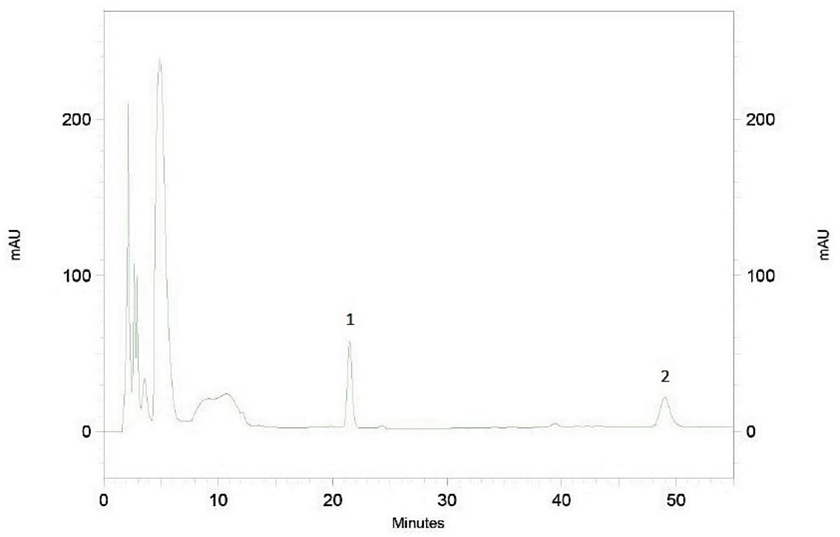

Figure S1. (**a**) HPLC chromatograms of standards solution; (**b**) methanolic extract of female bud of *C. sativa*: peak 1: cannabidiol (CBD), peak 2: Δ9-tetrahydrocannabinol (Δ9- THC).
